# Gene transcriptional profiles in gonads of *Bacillus* taxa (Phasmida) with different cytological mechanisms of automictic parthenogenesis

**DOI:** 10.1186/s40851-022-00197-z

**Published:** 2022-11-26

**Authors:** Giobbe Forni, Alexander S. Mikheyev, Andrea Luchetti, Barbara Mantovani

**Affiliations:** 1 Dip. Scienze Biologiche, Geologiche e Ambientali (BiGeA), University of Bologna, 40126 Bologna, Italy; 2 Dip. Scienze Agrarie e Ambientali, University of Milano, Milano, Italy; 3 Australian National University, ACT, Canberra, 2600 Australia; 4 Okinawa Institute of Science and Technology, 1919-1 Tancha, Onna-son, Okinawa, 904-0495 Japan

**Keywords:** dN/dS, Gene expression, Parthenogenesis, Phylostratigraphy, RNA-seq

## Abstract

The evolution of automixis – i.e., meiotic parthenogenesis – requires several features, including ploidy restoration after meiosis and maintenance of fertility. Characterizing the relative contribution of novel versus pre-existing genes and the similarities in their expression and sequence evolution is fundamental to understand the evolution of reproductive novelties. Here we identify gonads-biased genes in two *Bacillus* automictic stick-insects and compare their expression profile and sequence evolution with a bisexual congeneric species. The two parthenogens restore ploidy through different cytological mechanisms: in *Bacillus atticus*, nuclei derived from the first meiotic division fuse to restore a diploid egg nucleus, while in *Bacillus rossius*, diploidization occurs in some cells of the haploid blastula through anaphase restitution*.* Parthenogens’ gonads transcriptional program is found to be largely assembled from genes that were already present before the establishment of automixis. The three species transcriptional profiles largely reflect their phyletic relationships, yet we identify a shared core of genes with gonad-biased patterns of expression in parthenogens which are either male gonads-biased in the sexual species or are not differentially expressed there. At the sequence level, just a handful of gonads-biased genes were inferred to have undergone instances of positive selection exclusively in the parthenogen species. This work is the first to explore the molecular underpinnings of automixis in a comparative framework: it delineates how reproductive novelties can be sustained by genes whose origin precedes the establishment of the novelty itself and shows that different meiotic mechanisms of reproduction can be associated with a shared molecular ground plan.

**Supplementary Information:**

The online version contains supplementary material available at 10.1186/s40851-022-00197-z.

## Background

Parthenogenesis provides several advantages, including the transmission of beneficial allele combinations to future generations, improved colonization ability and reproductive success under environmental conditions with scarce mating opportunities [1, 2]. Despite being often associated with loss of heterozygosity due to the lack of outcrossing [3, 4], its importance as an adaptive trait is highlighted by the many independent times it has evolved across the tree of life [5]. In parthenogenetically reproducing females, ploidy maintenance can take place either through apomixis - with a mitotic process retaining the ploidy – or through automixis – in which meiosis is maintained, but a different set of mechanisms allow ploidy restoration [6]. The latter process can be achieved either by pre-meiotic extra-doubling of chromosome sets or by the fusion of meiotic products, which can happen through central fusion – i.e., restitutional meiosis at anaphase I or the fusion of its products – or through terminal fusion – i.e., restitutional meiosis at anaphase II or the fusion of its products [7]. Independently from the mechanism through which egg ploidy is either retained or restored, the establishment of parthenogenesis incurs additional and shared barriers, such as the activation of the oocyte without sperm fertilization [8]. Several clues to the causes and consequences of parthenogenesis in animals are available [9–14], but few studies leverage a solid knowledge on the modifications of the meiotic processes [15]. Investigations on well characterized automictic species have mainly focused on the consequences of this reproductive strategy [16–18] while an understanding of its molecular underpinnings is restricted to few taxa [19, 20].

**Fig. 1:**
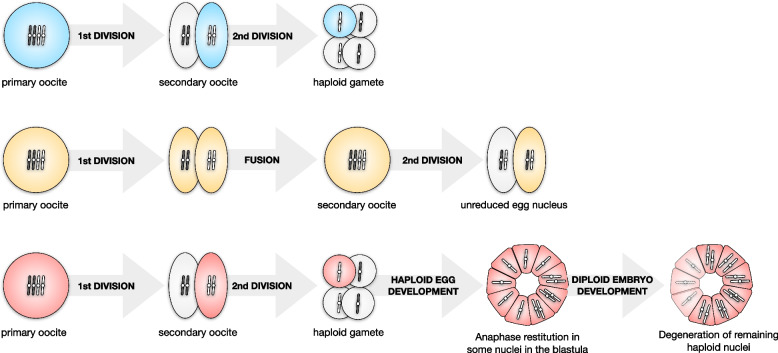
Overview of the oogenesis processes in **a** the bisexual *Bacillus grandii maretimi* (blue) and in the two parthenogens, **b** *Bacillus atticus* (yellow) and **c** *Bacillus rossius rossius* (red)

**Fig. 2:**
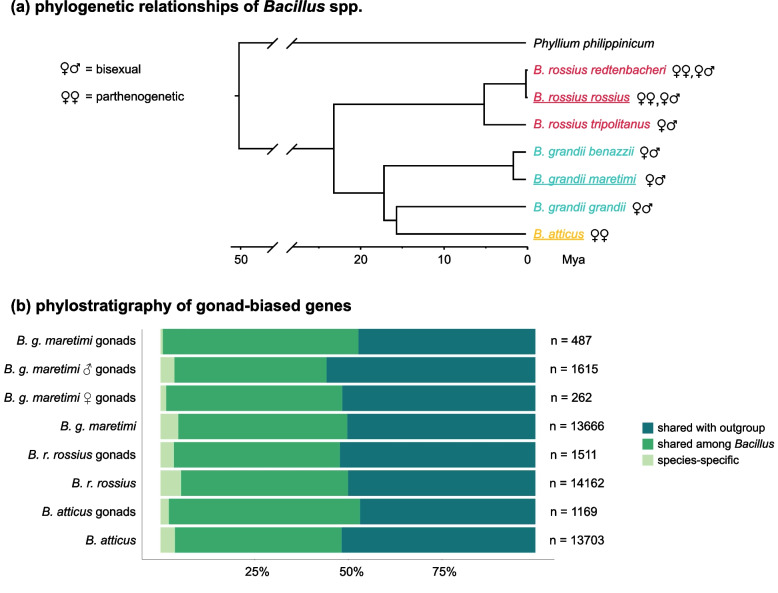
Phylogenetic relationships of *Bacillus* taxa and phylostratigraphy of gonads-biased genes. **a** Schematic drawing of the species tree obtained from Mantovani et al. (2001), with indication of the reproductive strategy for each taxon. Underlined species are those considered in the present study. **b** Phylostratigraphy of gonads upregulated genes; numbers on the right represent the number of transcripts present in each subset / assembly

Parthenogenesis is a well-known phenomenon in stick- and leaf-insects of the order Phasmida and it has been extensively characterized in the Mediterranean *Bacillus* stick insect species complex (Fig. 1) [21]; the latter started its diversification over 20 million years ago (Fig. 2a) [21, 22]. In this work we analyzed three *Bacillus* species of non-hybrid origin: *Bacillus rossius*, *Bacillus grandii* and *Bacillus atticus*. *Bacillus rossius* is a facultative parthenogenetic taxon with at least eight subspecies in the Mediterranean area: two South European subspecies, *B. rossius rossius* and *B. rossius redtenbacheri*, consist of both parthenogenetic and bisexual populations, while the North African and Spanish lineages comprise only bisexual populations. *Bacillus grandii* is a paraphyletic taxon which includes three obligate bisexual subspecies – *B. grandii grandii*, *B. grandii benazzii* and *B. grandii maretimi* – distributed in the Sicilian area. Finally, *B. atticus* is an obligate parthenogenon, whose all-female populations are distributed from Central to Eastern Mediterranean coasts, and it is the sister taxon of the bisexual *B. grandii grandii*. Earlier research provided evidence that the egg of *B. rossius* in parthenogenetic populations undergoes a canonical meiosis, but during the embryonic development some cells of the haploid blastula achieve diploidization via anaphase restitution (Fig. 1c); if fertilized, parthenogenetic *B. rossius* females may therefore reproduce bisexually giving rise to fertile offspring of both sexes [23]. In the obligate parthenogenon *B. atticus*, after a regular first meiotic division, nuclei at prophase II fuse in a diploid egg nucleus; this is followed by a second division leading to a degenerating polocyte and a quickly dividing, unreduced nucleus originating the parthenogenetic embryo (Fig. 1b) [24]. Although extant *B. atticus* populations are made by only females across the entire geographical range, studies on hybrid taxa suggested that males should have been present at least until 1 million years ago [22]. It is to be noted that the *B. rossius* automictic mechanism leads to a completely homozygous offspring, while the *B. atticus* one allows a certain degree of heterozygosity depending on chiasma position and chromosome segregation.

A key aspect to understand the mechanisms underlying evolutionary innovations is the relative contribution of novel genes versus pre-existing ones [25–27]. Both mechanisms can concur at the same time, leading to the evolution of novelties [28–30]. Nonetheless, the process through which novel traits are established can have different signatures, with some apparently linked to the appearance of novel genes [31, 32] and others more strongly associated with genes whose origin precedes the establishment of the novelty [33, 34]. In the latter case, the novelty can be associated to modifications of cis-regulatory elements affecting patterns of gene expression [35] and/or of protein coding sequence portions of genes [36, 37]. In this context, the degree of similarity shared by the molecular ground plan of analogous phenotypical endpoints is a key aspect [38, 39]. While the establishment of some traits seems to have happened through similar trajectories [40–42], in other cases evidence of such similar trajectories is lacking – implying that analogous outcomes are achieved through different paths [43, 44].

Here, we leveraged new transcriptomics resources of parthenogenetic *B. rossius* and *B. atticus,* and of the bisexual congeneric *B. grandii* to carry out comparative analyses of gene expression and molecular evolution, trying to elucidate: (1) the relative contribution to the evolution of automixis of pre-existing genes versus the emergence of novel ones, (2) the degree of similarity among parthenogen’s gonads transcriptional programs and (3) the genes which underwent instances of positive selection exclusively in parthenogens. Our analyses represent the first attempt to explore the transcriptional scheme associated with different automictic mechanisms, and the results from this work establish the *Bacillus* species complex as a new testing system to explore causes and consequences of these reproductive mechanisms.

## Methods

### Study design, RNA extraction and sequencing

*Bacillus grandii maretimi* (Marettimo Island, Sicily), *B. atticus* (Necropoli di Camarina, Sicily) and *B. rossius rossius* (Massa San Nicola, Sicily) specimens were collected in the field and lab-reared at 23 °C, feeding them with lentisk and bramble. Samples for the present analyses were isolated after 6 years of lab rearing. *Bacillus grandii maretimi* males were sacrificed 1 day after their final moult and females from all the three species within 24 h from their first egg laying, to ensure individuals were reproductively active and that reproductive structures were completely developed. Females of the bisexual taxon *B. grandii maretimi* were virgin. For female gonads, oviduct and accessory glands were removed and the tissue consisted of ovarioles containing a mixture of oocytes at different stages; for male gonads male testicle pairs were collected. Six biological replicates were collected, which consisted of (a) three legs from each individual for non-reproductive tissue (one foreleg, one mid-leg and one hind-leg) and (b) ovaries in females and testes in males for reproductive tissue; the same individual was used for leg and reproductive tract samples. Tissues were conserved in Trizol (Life Technologies) and RNA was extracted following the manufacturer’s protocol. RNA quantity and quality were measured using NanoDrop (Thermo Scientific) and Bioanalyzer (Agilent). Library preparation and paired-end sequencing (101 bp, NovaSeq 6000) were performed by Macrogen, Korea.

### Transcriptome assembly, orthology inference and phylostratigraphy

All available reads for each species were pooled and used for de novo assembly using Trinity [45] (Grabherr et al., 2011) with default parameters. We used TransDecoder (v5.5.0) on the raw assemblies to detect coding regions and UTRs in assembled transcripts, also integrating homology information inferred using blastp [46] against the SwissProt protein database and HMMER [47] against Pfam domain database (both downloaded in November 2021). When multiple ORFs were predicted for a transcript, we chose the single best one. To filter out of our assemblies all the transcripts which could be of contaminant origin, we carried out a blastp search of each species’ proteome against the database using an e-value cut-off of 1e-3 and subsequently assigned each hit to a specific lineage using TaxonKit [48]. We retained only those transcripts which had all of the 20 best hits as Metazoans and at least half of them as Panarthropoda. We excluded all of the transcripts which did not have any hit over the e-value cut-off. To provide a comparative framework across species, we carried out an inference to identify homologous gene clusters – including ortholog and paralog – using Orthofinder2 [49]. We included the translated proteomes of the three *Bacillus* species along with that of *Phyllium philippinicum* (TSA accession: GCPM00000000) [50] as an outgroup; the latter was chosen since it was found in a sister relationship with *B. rossius rossius* in a recent phylogenomic publication, where the divergence between the two clades was estimated to have happened around 50 mya [51]. A species tree was inferred according to the STAG method [52].

Subsequently, we leveraged the orthology inference to estimate the evolutionary age of genes using phylostratigraphy [53]; if a species shared a homolog with any of the other species, we assumed that the last common ancestor of the two already possessed a copy of this gene. Using a custom script, we defined for each species: (1) species-specific genes, as those which were found in orthogroups consisting of sequences only of the species considered (also known as Taxon Restricted Genes or orphan genes); (2) genes shared among *Bacillus*, as those which were found in orthogroups with at least one CDS of another *Bacillus* species, but lacking any *Phyllium* sequence; (3) genes shared with the outgroup, as those which were found in orthogroups with a *Phyllium* sequence. For each species, we tested whether the proportion of species-specific genes with gonads-biased pattern of expression was smaller than for the whole transcriptome, using a two-proportions z-test in R [54].

### Cross-species gene expression analyses

Reads from each sample and biological replicate were mapped on the relative species reference transcriptome using Bowtie2 [55] with default parameters and RSEM was used for transcript quantification [56]. Transcripts Per Million (TPM) expression values were cross-sample normalized using the trimmed mean of M-values (TMM) normalization method [57] for visualization and PCA analyses. The latter were carried out separately on gonad and leg samples, also considering either all genes or only those with a tissue-biased profile of expression. Using the gene level raw counts obtained from RSEM we ran the differential gene expression analyses between reproductive (gonads) and non-reproductive (legs) tissues – separately for each species and sex – using Deseq2 with Benjamini and Hochberg’s method for multiple tests correction [58]. Subsequently, we gathered the FDR and LogFC values for each transcript based on the orthology inference results, using a custom script. Gonads-biased genes were defined: 1) in parthenogens as those that showed an FDR < 0.05 and a LogFC > 1 between reproductive and non-reproductive tissues, 2) in *B. grandii maretimi* as those that showed an FDR < 0.05 and a LogFC > 1 between reproductive and non-reproductive tissues, in both sexes. For *B. grandii maretimi* we also defined male gonads-biased and female gonads-biased genes as those which had an FDR < 0.05 and a LogFC > 1 in one sex and were either not differentially expressed or downregulated in the other sex. Shifts in expression patterns between the two parthenogens and the sexual species were visualized using Sankey plots implemented in ggplot2 [59].

### Positive selection inference

We screened the 2840 protein-coding genes inferred as one-to-one orthologs across the four taxa for signatures of positive selection on the branches leading to *B. atticus* and *B. rossius rossius*. Initially we aligned all orthogroups as amino acids using two local alignment algorithms – l-INS-i and g-INS-i – implemented in MAFFT [60]. The amino acid alignments were then retro translated to nucleotides using pal2nal [61], and Gblocks was used to exclude spurious signals coming from misalignments [62]. We then inferred instances of positive selection using the branch-site model implemented in codeml [63]; all codeml analyses were carried out using BASE [64]. Separate analyses were performed using each terminal branch of our phylogeny as the foreground. We inferred two models, (a) one which had model = 2 and NSsites = 2 with ω_2_ fixed at one and (b) the same model, with ω_2_ free to vary. All codeml analyses were carried out using the fixed species tree and with branch lengths inferred using RAxML with a codon-aware partitioning scheme and a GTR model [65].

To assess whether the model including positive selection was a better fit than the one which did not consider it, we compared the likelihoods of the two models using a Likelihood Ratio Test (LRT) with one degree of freedom; the resulting *p* values were corrected for multiple tests using Benjamini and Hochberg’s method and genes were considered as candidates for positive selection when FDR < 0.05. Analyses were repeated for the two datasets aligned with l-INS-i and g-INS-i strategies: only the genes with a consistent signal of positive selection – independently from the alignment methods – were considered for subsequent analysis. To gain further lines of evidence for a possible role of these genes in the parthenogenetic reproductive process, we only considered genes which had an average TMM-normalized TPM > 10 in gonads. We extracted per-gene and per-branch number of sites which were found to be under positive selection by Bayes Empirical Bayes test (*p* < 0.95) and retrieved (1) genes undergoing species-specific positive selection (i.e., which had at least one site under positive selection in a single parthenogen terminal branch and none in the other species) and/or (2) genes undergoing parallel positive selection (i.e., which had at least one site under positive selection in both parthenogen terminal branch and none in the other species). To further confirm that our results were not driven by the alignment strategy, the candidate genes undergoing parallel positive selection were aligned using the homology extension approach implemented in PSI-Coffee [66]. Codeml analyses were repeated as before and additionally we cross-checked the results obtained by codeml using the aBSREL (adaptive Branch-Site Random Effects Likelihood) approach [67] implemented in HyPHY [68].

### Gene overlaps and GO enrichment

Fisher’s exact test implemented in the R package GeneOverlap [69] was used to determine *p* and odds ratio between the gene groups having parthenogens’ species-specific or shared/parallel transcriptional patterns and sequence modification, in comparison to a genomic background which consisted of all the orthogroups which shared at least two *Bacillus* species (*n* = 15,972). Functional annotation of genes was carried out separately for each species through blastp searches against the Uniref database (e-value <1e–3), combined with HMMER searches against the Pfam database; subsequently we generated GO-terms using Argot 2.5 with a TotalScore > 200 [70]. Subsequently we gathered all GO-terms associated with each orthogroup across the three *Bacillus* taxa, collapsing multiple entries of the same term. Enrichment analyses were performed with the TopGO package in Bioconductor, using Fisher’s exact test and both *elim* and *weight* algorithms – which considered GO hierarchy – and a node size of 2 [71]. GO-terms were considered significantly enriched when *elim p* < 0.05; genes associated with enriched terms of interest were retrieved and further characterized using BLASTP and HMMER online implementations [46, 72].

## Results

### Orthology inference and phylostratigraphy of gonads-biased genes

Definitive assemblies had a comparable number of coding sequences (CDSs): 13,666 for *B. grandii maretimi*, 13,703 for *B. atticus* and 14,162 for *B. rossius rossius*. The orthology inference yielded 2840 orthogroups consisting of single copy genes shared by all four taxa (i.e., including the outgroup) and 5283 single copy genes shared among the three *Bacillus* taxa. Single-copy genes which were found across each parthenogen comparison with the sexual species were respectively 6329 for *B. atticus* and 6201 for *B. rossius rossius*. The species tree – inferred according to the STAG (Species Tree inference from All Genes) algorithm implemented in Orthofinder2 – is consistent with previous hypotheses on the clade systematic relationships (Supplementary Fig. S1; Fig. 2a).

Phylostratigraphy was used to estimate a measure of genes origin of each species, to understand to what extent the establishment of parthenogenesis is coupled with the evolution of novel genes (Fig. 2b). Our analyses revealed that gonads-biased genes were found to be composed of a smaller proportion of species-specific genes compared to that which could be observed in the complete transcriptome assemblies: this pattern was observed not only for *B. atticus* (gonad-biased: 2.2%; overall: 3.8%) and *B. rossius rossius* (gonad-biased: 3.6%; overall: 5.4%), but also for the male and female gonad-biased genes in the bisexual species (male gonad-biased: 3.7%; female gonad-biased: 1.5%; gonad-biased in both sexes: 0.6%; overall 4.8%). In both parthenogens and the bisexual species the proportion of species-specific genes with gonads-biased pattern of expression was smaller than the proportion of species-specific genes in the whole transcriptome (two-proportions z-test *p* < 0.005).

### Shared and species-specific patterns of gonads gene expression across automictic species

**Fig. 3:**
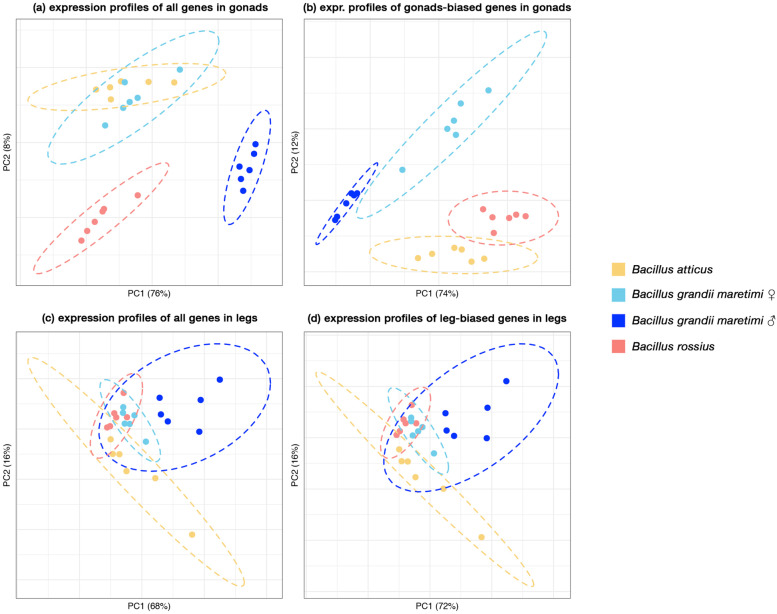
PCA analysis on the TMM normalized gene expression values for **a** all genes in gonads samples, **b** gonads-biased genes in gonads samples, **c** all genes in legs samples, (b) legs-biased genes in legs samples

**Fig. 4:**
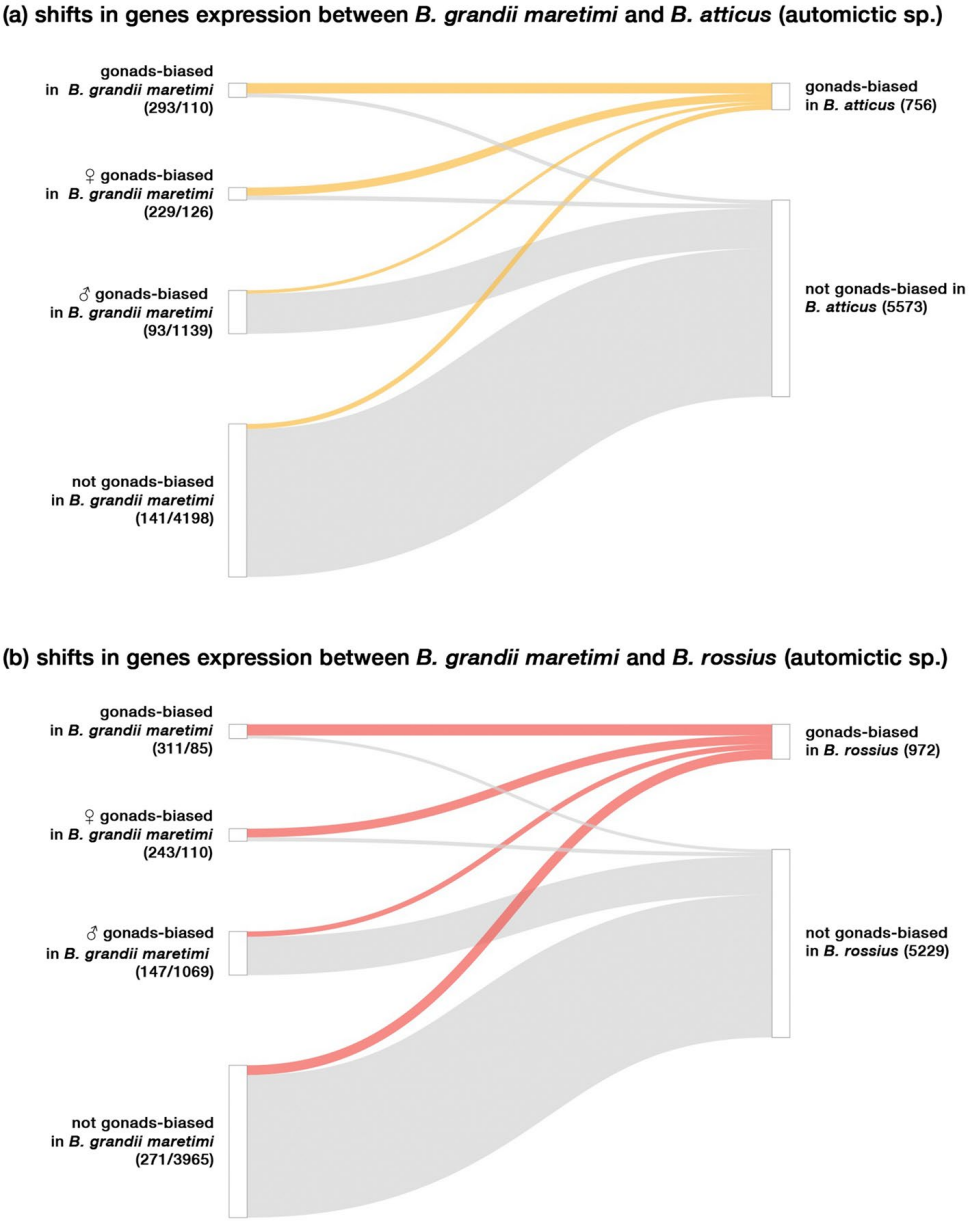
Gene expression patterns of parthenogens gonads compared to the bisexual species ones: **a** comparison between the automictic *Bacillus rossius rossius* and the bisexual *Bacillus grandii maretimi*, **b** comparison between the automictic *Bacillus atticus* and the bisexual *Bacillus grandii maretimi*. Numbers in parentheses are the genes with gonads-biased or non-biased expression regulation, respectively

To investigate similarities in gene expression patterns across parthenogen gonads with respect to the bisexual species, we leveraged two approaches: (1) a PCA analysis on the normalized counts (Fig. 3); (2) cross-species differential gene expression analyses (Fig. 4).

PCA analyses performed on all genes shared across the three *Bacillus* species clearly separated *B. grandii maretimi* male gonads from all other gonad samples, highlighting a general pattern of gene expression similarities among female gonads with respect to the male ones. For female gonads, the pattern found appears coherent with the phyletic relationships in the clade, separating *B. rossius rossius* from the more closely related *B. grandii maretimi* and *B. atticus*. When the PCA is carried out only on genes showing a gonad-biased profile of expression, the two parthenogenetic samples appear more similar to each other. Interestingly, when considering the gene expression in legs, the PCA pattern does not appear to be altered when considering only tissue-biased genes.

**Fig. 5:**
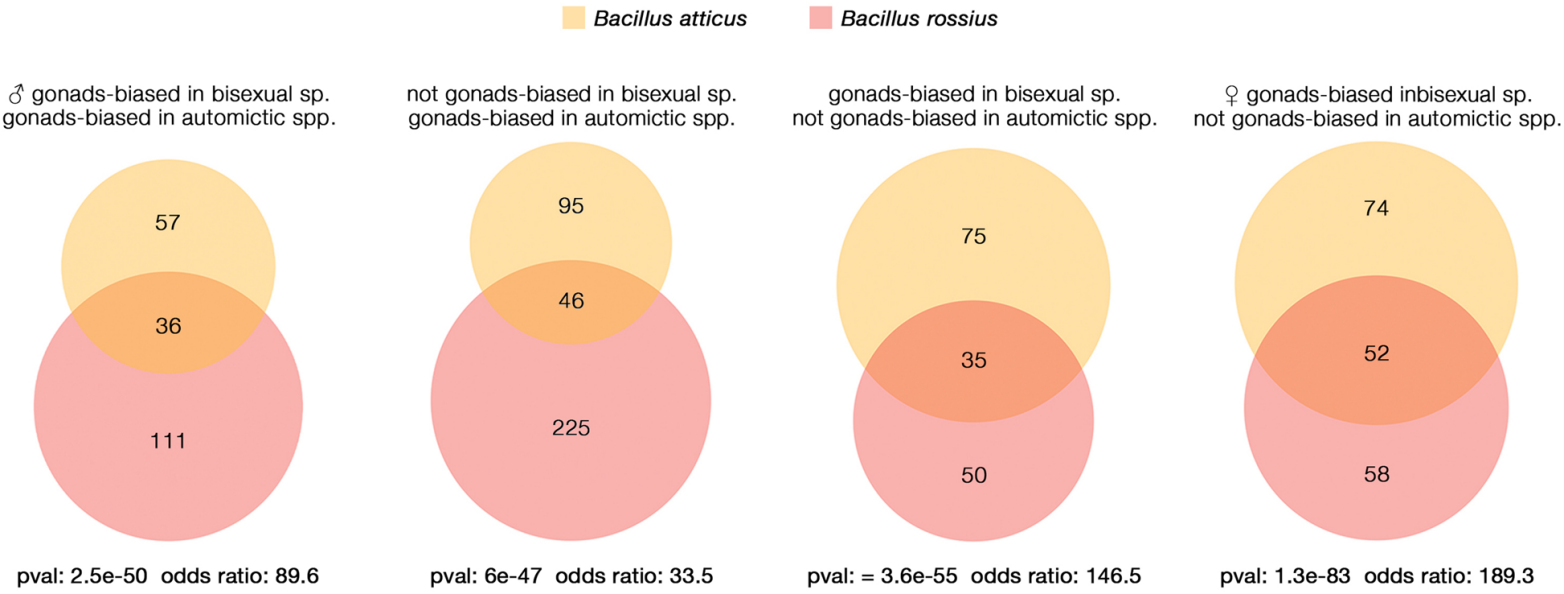
Venn-diagram representation of gonads genes expression patterns in comparison to the bisexual species. Overlaps represent parthenogens’ genes with a shared pattern in the two parthenogens, for which Fisher’s exact test has been used to determine *p* and odds ratio in comparison to the genomic background (*n* = 15,972)

Genes with gonads-biased expression in parthenogens appeared to be mainly composed of either gonads-biased (*B. atticus* = 293; *B. rossius rossius* = 311) or female gonads-biased (*B. atticus* = 229; *B. rossius rossius* = 243) in the bisexual taxon *B. grandii maretimi*. Nonetheless, we also retrieved genes which are either male gonads-biased (*B. atticus* = 93; *B. rossius rossius* = 147) or not gonads-biased in the bisexual species (*B. atticus* = 141; *B. rossius rossius* = 271). Moreover, some gonads-biased (*B. atticus* = 110; *B. rossius rossius* = 85) and female gonads-biased (*B. atticus* = 126; *B. rossius rossius* = 110) genes in the bisexual species were not found to have gonads-biased patterns of expression in parthenogens (Fig. 4). As patterns of gene expression can underlie lineage-specific adaptations or be the result of stochastic changes, we tested whether the overlap across the two interspecific comparisons, *B. atticus* vs *B. grandii maretimi* and *B. rossius rossius* vs *B. grandii maretimi* (Fig. 5), was significant. As expected, we retrieved a substantial similarity for genes which showed gonads-biased (*p* = 0; odds ratio = 505.9) and those not showing gonads-biased (*p* = 0; odds ratio = 50.5) expression profiles in the bisexual and parthenogenetic species (Supplementary Fig. S2). Genes showing a female gonads-biased pattern of expression in the sexual species and in parthenogens largely overlapped across parthenogens (*p* = 4e–281; odds ratio = 548.7); interestingly, similarity was also found in parthenogens’ gonads-biased genes which are male gonads-biased in the bisexual species (*p* = 2e–50; odds ratio = 89.6). Genes which showed a gonads-biased expression in parthenogens but were not gonads-biased in bisexual species showed a significant overlap between the two parthenogens (*p* = 6e–47; odds ratio = 33.5), as did those which exhibited a gonads-biased expression pattern in gonads of either bisexual females or across both sexes and were not gonads-biased in parthenogens (respectively, *p* = 3.6e–55; odds ratio = 146.5 and *p* = 1.3e–83; odds ratio = 189.3).

Guided by the previously obtained knowledge on the different mechanisms of automixis, we used the results of the GO-terms enrichment to explore their possible physiological functions. In parthenogens, many gonads-biased genes, which are male gonads-biased in the bisexual species, appeared to be related to centrosome formation and males’ reproductive mechanisms (Supplementary Table S1): these include the homologs of *centrosomal protein of 152 kDa* (CEP152 in OG0009808 [73]) and *centromere protein J* (CENPJ/CPAP in OG0007643 [74]). We also retrieved a homolog to *trimethyllysine dioxygenase* (TMLHE in OG0008327), which encodes an enzyme of the carnitine biosynthesis pathway, related to sperm count and motility in mammals [75, 76]. Genes showing a gonads-biased expression in parthenogens, but which were not gonads-biased in the sexual species (Supplementary Table S2) included a homolog to *spermidine synthase* (SRM in OG0002943); spermidine is a polyamine, a class of molecules which are essential to male and female reproductive processes, embryo development, mating and fertilization efficiency [77, 78]. Genes which are not gonads-biased in parthenogens but are either gonads-biased or female gonads-biased in the bisexual species (Supplementary Tables S3 and S4) included genes related to glycans potentially involved in oogenesis and egg coating, such as a homolog to *mannosyl-oligosaccharide 1,2-alpha-mannosidase* and *galactose-1-phosphate uridylyltransferase* (found in MAN1A1 in OG0006579 and GALT in OG0004972 [79–82]). A gene homolog to *mitotic checkpoint protein* (BUB3, found in OG0005535) was also found among them: its product regulates chromosome segregation during oocyte meiosis, with the dual function of spindle-assembly checkpoint signalling and establishment of correct kinetochore-microtubule attachments [83]. Only in *B. atticus* we retrieved a gonads-biased gene showing a substantial homology with *Anoctamin 6* (TMEM16F in OG0002369), a phospholipid scramblase which is involved in endocytosis and which mediates cell-cell fusion in the human trophoblast [84, 85].

### Parallel and species-specific sequence modifications across automictic species

**Fig. 6:**
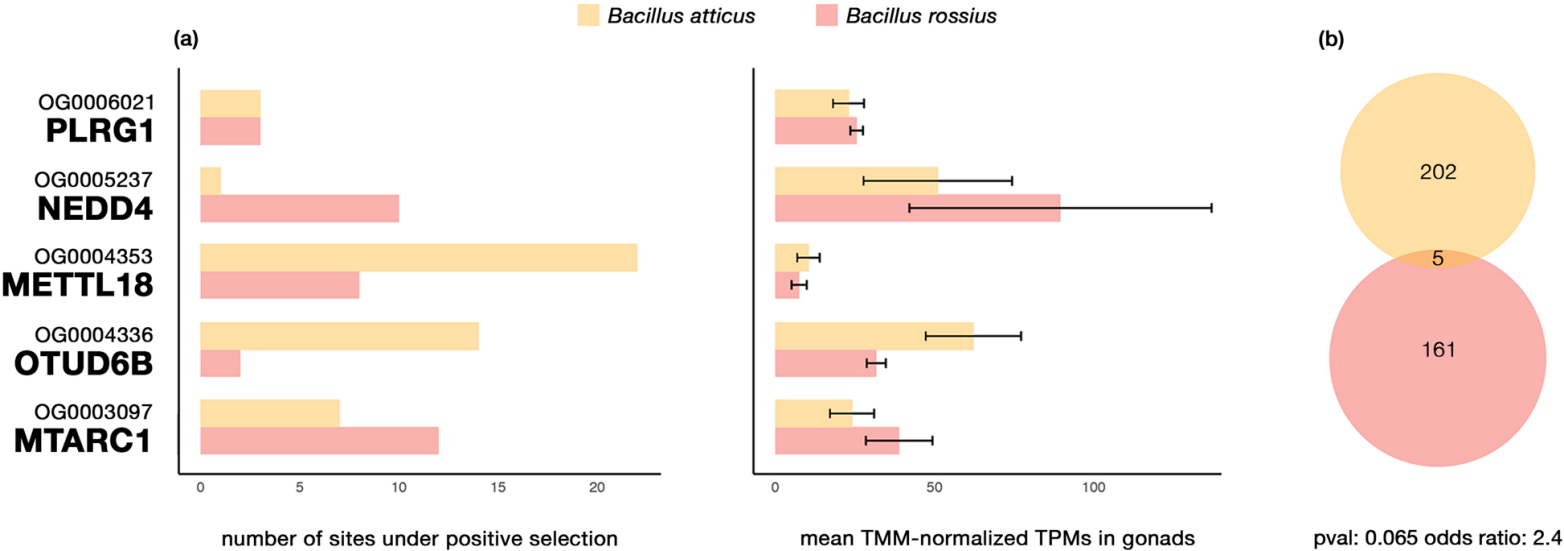
Parallel instances of positive selection: **a** on the left, number of sites inferred to have undergone positive selection (BEB > 95) in the two parthenogens; on the right, TMM-normalized expression values for the same genes. Genes with < 10 TPMs in parthenogen gonads were not considered. On the y axis, the orthogroups in which the genes are found, and the putative homologs (in bold) are given. **b** Venn-diagrams representation of the shared changes across the two parthenogens, for which Fisher’s exact test has been used to determine *p* and odds ratio in comparison to the genomic background (*n* = 15,972)

Using our species phylogeny, we explored instances of positive selection within a protein using branch-site codon models. Our screen identified 207 and 166 genes, respectively, in *B. rossius rossius* and *B. atticus*, which have at least one site under positive selection (*p* < 0.95 in the Bayes Empirical Bayes test) across the replicate analyses carried out using different local alignment strategies (g-INS-i and l-INS-i). To explore the degree of parallel sequence modifications affecting the same gene across the two automictic species we used stringent criteria: (1) we considered the impact of the alignment strategy on our results, and we filtered out possible misaligned regions; (2) we cross-checked with two different approaches (aBSREL and codeml); (3) we focused on signals shared exclusively among the parthenogenetic species and (4) we considered gonads’ gene expression. Six genes were found to have at least one site under positive selection in both parthenogens and not in the bisexual species within the codeml analyses, but one was not found to have undergone positive selection when cross-checked with aBSREL (Fig. 6; Supplementary Table S5). The overlap of genes undergoing instances of parallel positive selection across the two parthenogens was not significant (*p* = 0.65; odds ratio = 2.4).

As for modification of gene expression patterns, we leveraged the GO enrichment analyses to characterize genes inferred to have undergone species-specific sequence modifications (Supplementary Table S6). In *B. atticus* we retrieved genes homologous to *exocyst complex component 5* and *vacuolar protein sorting 39*, whose products are known to mediate vesicle trafficking and cytokinesis (respectively: EXOC5 in OG0002865 and VPS39 in OG0005132 [86, 87]. In *B. rossius rossius* we found two genes involved in spindle orientation: *dynactin subunit one* plays a role in metaphase spindle orientation and is required for microtubule anchoring at the mother centriole (DCTN1 in OG0003368 [88, 89]); *lethal(2) giant larvae protein* is involved in oocyte axis specification and in the regulation of mitotic spindle orientation through microtubule cytoskeleton organization (l(2) gl in G0005517 [90–93]). Among the five genes which underwent parallel sequence modifications, the products of *pleiotropic regulator 1*, *protein E3 ubiquitin-protein ligase NEDD4* and *OTU deubiquitinase 6B* homologs (PLRG1 in OG0006021; NEDD4 in OG0005237; OTUD6B in OG0004336) are all regulators involved in cell proliferation and differentiation in embryonic and reproductive tissues [94–97]. One homolog to *histidine protein methyltransferase 1* (METTL18 in OG0004353) has been identified in silico as a maternal factor potentially interacting with sperm factors [98]. *Mitochondrial amidoxime reducing component 1* (MTARC1 in OG0003097) gene encodes a signal-anchored protein of the outer mitochondrial membrane in humans [99].

## Discussion

The phylostratigraphic analyses showed that most of the genes with gonads-biased expression in parthenogens originated before the establishment of the novel reproductive strategy (Fig. 2b), consistent with patterns observed for other traits whose appearance is not clearly coupled with the de novo origination of genes [33, 34]. Since we relied on gene expression data, our analyses may have not included genes whose expression level is too low to be assembled; nonetheless, this potential bias is expected to similarly impact reproductive and non-reproductive tissues.

As reversions from parthenogenesis to sexuality have been proposed [100], it is not possible to make solid assumptions on the reproductive strategy of the common ancestor of the species considered. Therefore, whether parthenogenetic or bisexual, the observed gene expression similarities (Figs. 3 and 4) between the two parthenogens can be explained either as parallel changes from an ancestral state, which happened independently in the two lineages, or as the outcome of specific changes in the bisexual species only, coupled with the maintenance of the ancestral state in the two parthenogens. While it is tempting to interpret the observed pattern as similarly constrained or due to parallel evolutionary process associated with the establishment of automixis (Fig. 5), we cannot exclude a possible contribution of stochastic drift to the observed parthenogens’ gonads transcriptional patterns. It has been suggested, in fact, that changes in gonads gene expression can occur between congeneric species pairs in a short time, even without any change in the reproductive mode (between ~ 8% and ~ 15%) [101, 102]. While we had support for similarity at the transcriptional level, only five out of the thousands of genes showing gonads-biased expression analysed were found to have undergone parallel, positive selection in both parthenogens (Fig. 6), which contrasts with instances where evolutionary novelties were clearly associated with positive selection and convergent substitutions [40, 42, 103].

The physiological significance of some of the genes identified by our analyses can be associated with the restoration of ploidy without the male genetic contribution, such as for the homologs to *mitotic checkpoint protein*, *centromere protein J* and *centrosomal protein of 152 kDa* [104–107]. These genes could underlie a partially shared background for *Bacillus* in different automictic mechanisms and could represent either a novelty or an ancestral pre-adaptation for centrosomes assembly from maternal components without any paternal contribution [108]. Further species-specific features could then be associated with the different mechanisms through which ploidy is restored in the two species. Homologs to *exocyst complex component 5*, *vacuolar protein sorting 39* and *Anoctamin 6* were found to have undergone species-specific modifications in *B. atticus*: they are known to mediate vesicle trafficking/membrane fusion and could underlie the fusion of the nuclei produced by the first meiotic division [84, 86, 87, 95]. Furthermore, genes which could be involved in the anaphase restitution responsible for *B. rossius rossius* blastula diploidization are the *dynactin subunit one* and *lethal(2) giant larvae* homologs, which are involved in spindle orientation and oocyte axis specification; their modification could alter karyokinesis and cytokinesis in the blastula, impeding the separation of chromatids and resulting in restitution nuclei. Other than ploidy, an additional obstacle to the establishment of reproduction in the absence of males is represented by the maintenance of a high rate of oviposition in the absence of mating. The latter represents a strong physiological constraint, as highlighted by instances where males are maintained without any other clear genetic purpose [109]. Some of the genes which share an expression pattern exclusive to parthenogen gonads are associated with the production of molecules – such as carnitine or spermidine – which are present in male seminal fluids and have a role in fertilization or even act as pheromones in some animals [78, 110]. Regarding this aspect, an interesting gene which is inferred to have undergone parallel positive selection in the parthenogens is the homolog to *histidine methyltransferase*; this was found associated with developmental competence in oocytes and it has been identified in silico as a maternal factor potentially interacting with the sperm factor Hdac11 and thus with a potential role in processes downstream from the sperm-egg fusion [98, 111]. Nonetheless, not all parthenogen’s similarities are expected to concur with the establishment of automixis; many could also result from the removal of selective pressures on mating-associated mechanisms and their subsequent decay [12, 16, 112]. Interestingly, parthenogens lack the gonads-biased pattern of expression in genes related to the production of glycans involved in oogenesis and egg coating, possibly because the absence of sperm-egg interaction makes their maintenance unnecessary.

It is to be noted, though, that even if presently suggested functions proposed for these genes may hint to their involvement in the processes discussed, experimental and functional studies are needed to verify and confirm their actual role.

## Conclusions

Our analyses did not retrieve many genes with similar functions, but this is coherent with the observed association of reproductive strategies with few – or even single – genes, and further suggested that the switch towards a novel reproduction strategy can largely depend on changes in the expression of pre-existing genes [19, 20, 113–115]. The genes here reported have known functions in model species which may underlie the evolution of automixis in *Bacillus*; yet the phenotypic effects of their sequence and expression modification is unknown in the context of the species considered. At this stage, complementary functional studies are needed to confirm their possible role in automixis and a larger species sampling is necessary to disentangle stochastic changes from those generated by selective forces. Nonetheless, different novel and analogous automictic mechanisms are associated with a largely shared ground plan made by genes whose origin predates the shift in reproductive strategy.

## Supplementary Information

**Additional file 1:** **Supplementary Fig. S1.** Species tree obtained with STAG (Species Tree inference from All Genes) algorithm implemented in Orthofinder2. Reproductive strategies and the mechanisms of diploidy restoration are also reported; asterisks indicate the two independent shifts to parthenogenesis.

**Additional file 2: Supplementary Fig. S2.** Venn-diagrams represent the genes with different expression patterns between parthenogens and the bisexual species. Overlaps represent genes whose gonad pattern of expression is shared across the two parthenogens for which Fisher’s exact test has been used to determine *p* and odds ratio, in comparison to the genomic background (*n* = 15,972).

**Additional file 3: Supplementary Table S1.** GO-terms enrichment analysis for genes which are male gonads-biased in the bisexual species and gonads-biased in parthenogen’s gonads. Throughout the table sheets the following abbreviations are used: BRO for *Bacillus rossius rossius*, BAT for *Bacillus atticus*, BGM for *Bacillus grandii maretimi*, MF for Molecular Functions and BP for Biological Processes.

**Additional file 4:** **Supplementary Table S2.** GO-terms enrichment analysis for genes which are not gonads-biased in the bisexual species and gonads-biased in parthenogen’s gonads. The abbreviations used are the same as in the other Supplementary tables.

**Additional file 5:** **Supplementary Table S3.** GO-terms enrichment analysis for genes which are gonads-biased in the bisexual species and not gonads-biased in parthenogen’s gonads. The abbreviations used are the same as in the other Supplementary tables.

**Additional file 6:** **Supplementary Table S4.** GO-terms enrichment analysis for genes which are gonads-biased in bisexual species females and not gonads-biased in parthenogen’s gonads. The abbreviations used are the same as in the other Supplementary tables.

**Additional file 7:** **Supplementary Table S5.** Results of the branch-site analysis using codeml of the candidate genes for parallel sequence modifications. The analysis was carried out separately for each species and using the alignment generated by PSI-Coffee; additional cross-validation was carried out using aBSREL. The abbreviations used are the same as in the other Supplementary tables.

**Additional file 8:** **Supplementary Table S6.** GO-terms enrichment analysis of candidate genes for species-specific instances of positive selection in the two parthenogens. The abbreviations used are the same as in the other Supplementary tables.

## Data Availability

All RNA-seq reads are deposited on the SRA under accession numbers SRX7034623–SRX7034670 (Bioproject PRJNA578804), while reference transcriptomes have been uploaded to the TSA under accession GJDY01000000, GJDZ01000000, and GJEA00000000. All custom scripts associated with this project and intermediate files are deposited at (https://github.com/for-giobbe/gene-transcriptional-profiles-in-automictic-taxa).

## Abbreviations

UTR: Untranslated region
ORF: Open reading frame
CDS: Coding sequence
TPM: Transcript Per Million
TMM: trimmed mean of M-values
FDR: False discovery rate
GTR: General Time reversible
LRT: Likelihood Ratio Test
GO: Gene ontology

## References

[1] Lehtonen J, Jennions MD, Kokko H (2012). The many costs of sex. Trends Ecol Evol.

[2] Tilquin A, Kokko H (2016). What does the geography of parthenogenesis teach us about sex?. Phil Trans R Soc B..

[3] Engelstädter J (2017). Asexual but not clonal: evolutionary processes in automictic populations. Genetics..

[4] Svendsen N, Reisser CM, Dukić M, Thuillier V, Ségard A, Liautard-Haag C (2015). Uncovering cryptic asexuality in *Daphnia magna* by RAD sequencing. Genetics..

[5] Mogie M (1986). Automixis: its distribution and status. Biol J Linn Soc.

[6] Normark BB, Maloy S, Hughes K (2013). Parthenogenesis. Brenner's Encyclopedia of genetics (2nd edition).

[7] Mirzaghaderi G, Hörandl E (2016). The evolution of meiotic sex and its alternatives. Proc Roy Soc B..

[8] Pál C, Papp B (2017). Evolution of complex adaptations in molecular systems. Nat Ecol Evol.

[9] Bast J, Parker DJ, Dumas Z, Jalvingh KM, Tran Van P, Jaron KS (2018). Consequences of asexuality in natural populations: insights from stick insects. Mol Biol Evol.

[10] Jaron KS, Bast J, Nowell RW, Ranallo-Benavidez TR, Robinson-Rechavi M, Schwander T (2021). Genomic features of parthenogenetic animals. J Hered..

[11] Neiman M, Sharbel TF, Schwander T (2014). Genetic causes of transitions from sexual reproduction to asexuality in plants and animals. J Evol Biol.

[12] Parker DJ, Bast J, Jalvingh K, Dumas Z, Robinson-Rechavi M, Schwander T (2019). Repeated evolution of asexuality involves convergent gene expression changes. Mol Biol Evol.

[13] Parker DJ, Bast J, Jalvingh K, Dumas Z, Robinson-Rechavi M, Schwander T (2019). Sex-biased gene expression is repeatedly masculinized in asexual females. Nat Commun.

[14] Tvedte ES, Logsdon JM, Forbes AA (2019). Sex loss in insects: causes of asexuality and consequences for genomes. Curr Opin Insect Sci.

[15] Jaron KS, Parker DJ, Anselmetti Y, Tran Van P, Bast J, Dumas Z (2022). Convergent consequences of parthenogenesis on stick insect genomes. Sci Adv.

[16] Kraaijeveld K, Anvar SY, Frank J, Schmitz A, Bast J, Wilbrandt J (2016). Decay of sexual trait genes in an asexual parasitoid wasp. Genome Biol Evol..

[17] Matsuura K (2017). Evolution of the asexual queen succession system and its underlying mechanisms in termites. J Exp Biol.

[18] Tvedte ES, Forbes AA, Logsdon JM (2017). Retention of core meiotic genes across diverse Hymenoptera. J Hered.

[19] Wallberg A, Pirk CW, Allsopp MH, Webster MT (2016). Identification of multiple loci associated with social parasitism in honeybees. PLoS Genet.

[20] Yagound B, Dogantzis KA, Zayed A, Lim J, Broekhuyse P, Remnant EJ (2020). A single gene causes thelytokous parthenogenesis, the defining feature of the cape honeybee *Apis mellifera capensis*. Curr Biol.

[21] Scali V, Passamonti M, Marescalchi O, Mantovani B (2003). Linkage between sexual and asexual lineages: genome evolution in *Bacillus* stick insects. Biol J Linn Soc.

[22] Mantovani B, Passamonti M, Scali V (2001). The mitochondrial cytochrome oxidase II gene in *Bacillus* stick insects: ancestry of hybrids, androgenesis, and phylogenetic relationships. Mol Phylogenet Evol.

[23] Pijnacker LP (1969). Automictic parthenogenesis in the stick insect *Bacillus rossius* Rossi (Cheleutoptera, Phasmidae). Genetica..

[24] Marescalchi O, Pijnacker LP, Scali V (1993). Automictic parthenogenesis and its genetic consequence in *Bacillus atticus atticus* (Insecta Phasmatodea). Inv Repr Dev.

[25] McLysaght A, Guerzoni D (2015). New genes from non-coding sequence: the role of de novo protein-coding genes in eukaryotic evolutionary innovation. Phil Trans R Soc B.

[26] Shubin N, Tabin C, Carroll S (2009). Deep homology and the origins of evolutionary novelty. Nature..

[27] Tautz D, Domazet-Lošo T (2011). The evolutionary origin of orphan genes. Nat Rev Genet.

[28] Aguilera F, McDougall C, Degnan BM (2017). Co-option and de novo gene evolution underlie molluscan shell diversity. Mol Biol Evol.

[29] Albertin CB, Simakov O, Mitros T, Wang ZY, Pungor JR, Edsinger-Gonzales E (2015). The octopus genome and the evolution of cephalopod neural and morphological novelties. Nature..

[30] Hilgers L, Hartmann S, Hofreiter M, von Rintelen T (2018). Novel genes, ancient genes, and gene co-option contributed to the genetic basis of the radula, a molluscan innovation. Mol Biol Evol.

[31] Babonis LS, Martindale MQ, Ryan JF (2016). Do novel genes drive morphological novelty? An investigation of the nematosomes in the sea anemone *Nematostella vectensis*. BMC Evol Biol.

[32] Santos ME, Le Bouquin A, Crumière AJ, Khila A (2017). Taxon-restricted genes at the origin of a novel trait allowing access to a new environment. Science..

[33] Almudi I, Vizueta J, Wyatt CD, de Mendoza A, Marlétaz F, Firbas PN (2020). Genomic adaptations to aquatic and aerial life in mayflies and the origin of insect wings. Nat Commun.

[34] Jasper WC, Linksvayer TA, Atallah J, Friedman D, Chiu JC, Johnson BR (2015). Large-scale coding sequence change underlies the evolution of post developmental novelty in honeybees. Mol Biol Evol.

[35] McGirr JA, Martin CH (2021). Few fixed variants between trophic specialist pupfish species reveal candidate cis-regulatory alleles underlying rapid craniofacial divergence. Mol Biol Evol.

[36] Casewell NR, Petras D, Card DC, Suranse V, Mychajliw AM, Richards D (2019). Solenodon genome reveals convergent evolution of venom in eulipotyphlan mammals. Proc Natl Acad Sci U S A.

[37] Jebb D, Huang Z, Pippel M, Hughes GM, Lavrichenko K, Devanna P (2020). Six reference-quality genomes reveal evolution of bat adaptations. Nature..

[38] Sackton TB, Clark N (2019). Convergent evolution in the genomics era: new insights and directions. Phil Trans Roy S B.

[39] Stern DL (2013). The genetic causes of convergent evolution. Nat Rev Genet..

[40] Burskaia V, Naumenko S, Schelkunov M, Bedulina D, Neretina T, Kondrashov A (2020). Excessive parallelism in protein evolution of Lake Baikal amphipod species flock. Genome Biol Evol.

[41] Warner MR, Qiu L, Holmes MJ, Mikheyev AS, Linksvayer TA (2019). Convergent eusocial evolution is based on a shared reproductive ground plan plus lineage-specific plastic genes. Nat Commun.

[42] Yuan J, Zhang X, Gao Y, Zhang X, Liu C, Xiang J, Li F (2020). Adaptation and molecular evidence for convergence in decapod crustaceans from deep-sea hydrothermal vent environments. Mol Ecol.

[43] Corbett-Detig RB, Russell SL, Nielsen R, Losos J (2020). Phenotypic convergence is not mirrored at the protein level in a lizard adaptive radiation. Mol Biol Evol.

[44] Zou Z, Zhang J (2015). No genome-wide protein sequence convergence for echolocation. Mol Biol Evol.

[45] Grabherr MG, Haas BJ, Yassour M, Levin JZ, Thompson DA, Amit I (2011). Full-length transcriptome assembly from RNA-Seq data without a reference genome. Nat Biotechnol.

[46] Camacho C, Coulouris G, Avagyan V, Ma N, Papadopoulos J, Bealer K, Madden TL (2009). BLAST+: architecture and applications. BMC Bioinformatics.

[47] Finn RD, Clements J, Eddy SR (2011). HMMER web server: interactive sequence similarity searching. Nucleic Acids Res.

[48] Shen W, Xiong J (2021). TaxonKit: a cross-platform and efficient NCBI taxonomy toolkit. J Genet Genom.

[49] Emms DM, Kelly S (2019). OrthoFinder: phylogenetic orthology inference for comparative genomics. Genome Biol.

[50] Wipfler B, Letsch H, Frandsen PB, Kapli P, Mayer C, Bartel D (2019). Evolutionary history of Polyneoptera and its implications for our understanding of early winged insects. Proc Natl Acad Sci U S A.

[51] Simon S, Letsch H, Bank S, Buckley TR, Donath A, Liu S (2019). Old World and New World phasmatodea: phylogenomics resolve the evolutionary history of stick and leaf insects. Front Ecol Evol.

[52] Emms DM, Kelly S. STAG: Species Tree Inference from All Genes. bioRxiv. 2018:267914. 10.1101/267914.

[53] Domazet-Lošo T, Brajković J, Tautz D (2007). A phylostratigraphy approach to uncover the genomic history of major adaptations in metazoan lineages. Trends Genet.

[54] R Core Team. R: a language and environment for statistical computing. Vienna: R Foundation for Statistical Computing; 2022. https://www.R-project.org/. Accessed Sept 2022.

[55] Langmead B, Salzberg SL (2012). Fast gapped-read alignment with bowtie 2. Nat Methods.

[56] Li B, Dewey CN (2011). RSEM: accurate transcript quantification from RNA-Seq data with or without a reference genome. BMC Bioinformatics..

[57] Robinson MD, Oshlack A (2010). A scaling normalization method for differential expression analysis of RNA-seq data. Genome Biol.

[58] Love MI, Huber W, Anders S (2014). Moderated estimation of fold change and dispersion for RNA-seq data with DESeq2. Genome Biol.

[59] Wickham H (2016). ggplot2: elegant graphics for data analysis.

[60] Katoh K, Standley DM (2013). MAFFT multiple sequence alignment software version 7: improvements in performance and usability. Mol Biol Evol.

[61] Suyama M, Torrents D, Bork P (2006). PAL2NAL: robust conversion of protein sequence alignments into the corresponding codon alignments. Nuc Acids Res..

[62] Talavera G, Castresana J (2007). Improvement of phylogenies after removing divergent and ambiguously aligned blocks from protein sequence alignments. Syst Biol.

[63] Zhang J, Nielsen R, Yang Z (2005). Evaluation of an improved branch-site likelihood method for detecting positive selection at the molecular level. Mol Biol Evol.

[64] Forni G, Ruggeri AA, Piccinini G, Luchetti A (2021). BASE: a novel workflow to integrate non-ubiquitous genes in genomics analyses for selection. Ecol Evol.

[65] Stamatakis A (2014). RAxML version 8: a tool for phylogenetic analysis and post-analysis of large phylogenies. Bioinformatics..

[66] Chang JM, Di Tommaso P, Taly JF, Notredame C (2012). Accurate multiple sequence alignment of transmembrane proteins with PSI-coffee. BMC Bioinformatics..

[67] Smith MD, Wertheim JO, Weaver S, Murrell B, Scheffler K, Kosakovsky Pond SL (2015). Less is more: an adaptive branch-site random effects model for efficient detection of episodic diversifying selection. Mol Biol Evol.

[68] Pond SLK, Muse SV, Nielsen R (2005). HyPhy: hypothesis testing using phylogenies. Statistical methods in molecular evolution. Statistics for biology and health.

[69] Shen L, Sinai M. GeneOverlap: Test and visualize gene overlaps. R package version 1.26.0. 2020. http://shenlab-sinai.github.io/shenlab-sinai/. Accessed 15 Jan 2022.

[70] Lavezzo E, Falda M, Fontana P, Bianco L, Toppo S (2016). Enhancing protein function prediction with taxonomic constraints–the Argot2. 5 web server. Methods..

[71] Alexa A, Rahnenführer J (2009). Gene set enrichment analysis with topGO. Bioconductor Improvement.

[72] Potter SC, Luciani A, Eddy SR, Park Y, Lopez R, Finn RD (2018). HMMER web server: 2018 update. Nuc Acids Res.

[73] Kodani A, Yu TW, Johnson JR, Jayaraman D, Johnson TL, Al-Gazali L (2015). Centriolar satellites assemble centrosomal microcephaly proteins to recruit CDK2 and promote centriole duplication. Elife..

[74] Chang J, Cizmecioglu O, Hoffmann I, Rhee K (2010). PLK2 phosphorylation is critical for CPAP function in procentriole formation during the centrosome cycle. EMBO J.

[75] Ng CM, Blackman MR, Wang C, Swerdloff RS (2004). The role of carnitine in the male reproductive system. Ann N Y Acad Sci.

[76] Poiani A (2006). Complexity of seminal fluid: a review. Behav Ecol Sociobiol.

[77] Bauer MA, Carmona-Gutiérrez D, Ruckenstuhl C, Reisenbichler A, Megalou EV, Eisenberg T (2013). Spermidine promotes mating and fertilization efficiency in model organisms. Cell Cycle.

[78] Lefèvre PL, Palin MF, Murphy BD (2011). Polyamines on the reproductive landscape. Endocrine Rev.

[79] Akintayo AA, Stanley P (2019). Roles for Golgi glycans in oogenesis and spermatogenesis. Front Cell Dev Biol.

[80] Avilés M, Okinaga T, Shur BD, Ballesta J (2000). Differential expression of glycoside residues in the mammalian zona pellucida. Mol Rep Dev.

[81] Cornwall GA, Tulsiani DRP, Orgebin-Crist MC (1991). Inhibition of the mouse sperm surface α-D-mannosidase inhibits sperm-egg binding in vitro. Biol Reprod.

[82] Nishimura K, Dioguardi E, Nishio S, Villa A, Han L, Matsuda T, Jovine L (2019). Molecular basis of egg coat cross-linking sheds light on ZP1-associated female infertility. Nat Commun.

[83] Kalitsis P, Earle E, Fowler KJ, Choo KA (2000). Bub3 gene disruption in mice reveals essential mitotic spindle checkpoint function during early embryogenesis. Genes Dev.

[84] Bricogne C, Fine M, Pereira PM, Sung J, Tijani M, Wang Y (2019). TMEM16F activation by Ca^2+^ triggers plasma membrane expansion and directs PD-1 trafficking. Sci Rep.

[85] Zhang Y, Le T, Grabau R, Mohseni Z, Kim H, Natale DR (2020). TMEM16F phospholipid scramblase mediates trophoblast fusion and placental development. Sci Adv.

[86] Richardson SC, Winistorfer SC, Poupon V, Luzio JP, Piper RC (2004). Mammalian late vacuole protein sorting orthologues participate in early endosomal fusion and interact with the cytoskeleton. Mol Biol Cell.

[87] Wang H, Tang X, Liu J, Trautmann S, Balasundaram D, Mccollum D, Balasubramanian MK (2002). The multiprotein exocyst complex is essential for cell separation in Schizosaccharomyces pombe. Mol Biol Cell.

[88] Kiyomitsu T, Cheeseman IM (2012). Chromosome-and spindle-pole-derived signals generate an intrinsic code for spindle position and orientation. Nat Cell Biol.

[89] Kodani A, Sirerol-Piquer MS, Seol A, Garcia-Verdugo JM, Reiter JF (2013). Kif3a interacts with dynactin subunit p150Glued to organize centriole subdistal appendages. EMBO J.

[90] Albertson R, Doe CQ (2003). Dlg, Scrib and Lgl regulate neuroblast cell size and mitotic spindle asymmetry. Nat Cell Biol.

[91] Bilder D, Li M, Perrimon N (2000). Cooperative regulation of cell polarity and growth by *Drosophila* tumor suppressors. Science..

[92] Carvalho CA, Moreira S, Ventura G, Sunkel CE, Morais-de-Sá E (2015). Aurora a triggers Lgl cortical release during symmetric division to control planar spindle orientation. Curr Biol.

[93] Li Q, Xin T, Chen W, Zhu M, Li M (2008). Lethal (2) giant larvae is required in the follicle cells for formation of the initial AP asymmetry and the oocyte polarity during *Drosophila* oogenesis. Cell Res.

[94] Kleinridders A, Pogoda HM, Irlenbusch S, Smyth N, Koncz C, Hammerschmidt M, Brüning JC (2009). PLRG1 is an essential regulator of cell proliferation and apoptosis during vertebrate development and tissue homeostasis. Mol Cell Biol.

[95] Sakata T, Sakaguchi H, Tsuda L, Higashitani A, Aigaki T, Matsuno K, Hayashi S (2004). Drosophila Nedd4 regulates endocytosis of notch and suppresses its ligand-independent activation. Curr Biol.

[96] Sobol A, Askonas C, Alani S, Weber MJ, Ananthanarayanan V, Osipo C, Bocchetta M (2017). Deubiquitinase OTUD6B isoforms are important regulators of growth and proliferation. Mol Cancer Res.

[97] Zhou Z, Kawabe H, Suzuki A, Shinmyozu K, Saga Y (2017). NEDD4 controls spermatogonial stem cell homeostasis and stress response by regulating messenger ribonucleoprotein complexes. Nat Commun.

[98] Ntostis P, Carter D, Iles D, Huntriss J, Tzetis M, Miller D (2017). Potential sperm contributions to the murine zygote predicted by in silico analysis. Reproduction..

[99] Klein JM, Busch JD, Potting C, Baker MJ, Langer T, Schwarz G (2012). The mitochondrial amidoxime-reducing component (mARC1) is a novel signal-anchored protein of the outer mitochondrial membrane. J Biol Chem.

[100] Domes K, Norton RA, Maraun M, Scheu S (2007). Reevolution of sexuality breaks Dollo's law. Proc Natl Acad Sci U S A.

[101] Lopez-Maestre H, Carnelossi EA, Lacroix V, Burlet N, Mugat B, Chambeyron S (2017). Identification of misexpressed genetic elements in hybrids between *Drosophila*-related species. Sci Rep.

[102] Ponnanna K, DSouza SM, Amruthavalli C, Ramachandra NB. (2021). Allopatric sibling species pair *Drosophila nasuta nasuta* and *Drosophila nasuta albomicans* exhibit expression divergence in ovarian transcriptomes. Gene..

[103] Wang Y, Yang L (2021). Genomic evidence for convergent molecular adaptation in electric fishes. Genome Biol Evol..

[104] Cho JH, Chang CJ, Chen CY, Tang TK (2006). Depletion of CPAP by RNAi disrupts centrosome integrity and induces multipolar spindles. Biochem Biophys Res Comm.

[105] Kohlmaier G, Lončarek J, Meng X, McEwen BF, Mogensen MM, Spektor A (2009). Overly long centrioles and defective cell division upon excess of the SAS-4-related protein CPAP. Curr Biol.

[106] Dzhindzhev NS, Quan DY, Weiskopf K, Tzolovsky G, Cunha-Ferreira I, Riparbelli M (2010). Asterless is a scaffold for the onset of centriole assembly. Nature..

[107] Lee M, Chang J, Chang S, Lee KS, Rhee K (2014). Asymmetric spindle pole formation in CPAP-depleted mitotic cells. Biochem Biophys Res Comm..

[108] Marescalchi O, Zauli C, Scali V (2002). Centrosome dynamics and inheritance in related sexual and parthenogenetic *Bacillus* (Insecta Phasmatodea). Mol Rep Dev..

[109] Miyakawa MO, Mikheyev AS (2015). Males are here to stay: fertilization enhances viable egg production by clonal queens of the little fire ant (*Wasmannia auropunctata*). Sci Nat.

[110] Scott AM, Zhang Z, Jia L, Li K, Zhang Q, Dexheimer T (2019). Spermine in semen of male sea lamprey acts as a sex pheromone. PLoS Biol.

[111] Biase FH, Kimble KM (2018). Functional signaling and gene regulatory networks between the oocyte and the surrounding cumulus cells. BMC Genomics.

[112] Schwander T, Crespi BJ, Gries R, Gries G (2013). Neutral and selection-driven decay of sexual traits in asexual stick insects. Proc Roy Soc B..

[113] Ma W-J, Pannebakker BA, Li X, Geuverink E, Anvar SY, Veltsos P (2021). A single QTL with large effect is associated with female functional virginity in an asexual parasitoid wasp. Mol Ecol.

[114] Huylmans AK, Macon A, Hontoria F, Vicoso B (2021). Transitions to asexuality and evolution of gene expression in *Artemia* brine shrimp. Proc Roy Soc B.

[115] Bartoš O, Röslein J, Kotusz J, Paces J, Pekárik L, Petrtýl M (2019). The legacy of sexual ancestors in phenotypic variability, gene expression, and homoeolog regulation of asexual hybrids and polyploids. Mol Biol Evol.

