# Supplementary figures and images for: Gene transcriptional profiles in gonads of Bacillus taxa (Phasmida) with different cytological mechanisms of automictic parthenogenesis
Source: Zoological Lett. 2022 Nov 26;8:14. doi: 10.1186/s40851-022-00197-z (PMC9701443; doi:10.1186/s40851-022-00197-z)

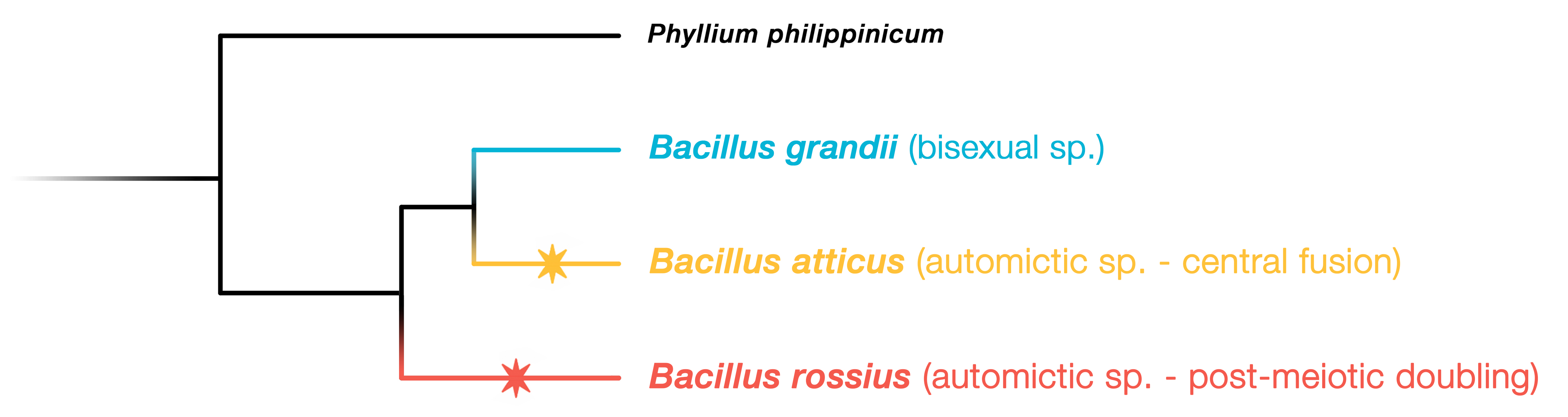

Supplement: Supplementary file 1 — Additional file 1: Supplementary Fig. S1. Species tree obtained with STAG (Species Tree inference from All Genes) algorithm implemented in Orthofinder2. Reproductive strategies and the mechanisms of diploidy restoration are also reported; asterisks indicate the two independent shifts to parthenogenesis. [file 40851_2022_197_MOESM1_ESM.png]

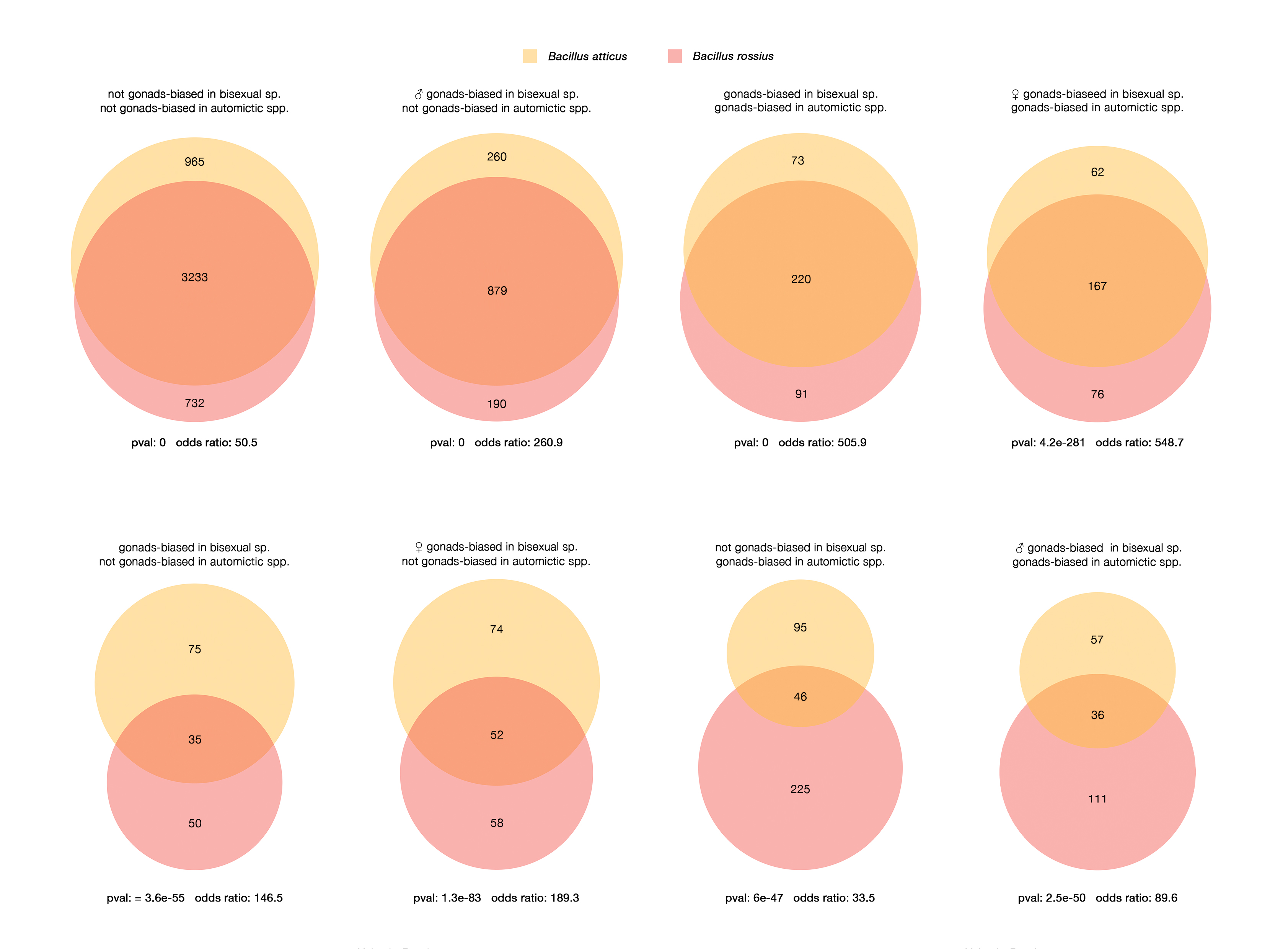

Supplement: Supplementary file 2 — Additional file 2: Supplementary Fig. S2. Venn-diagrams represent the genes with different expression patterns between parthenogens and the bisexual species. Overlaps represent genes whose gonad pattern of expression is shared across the two parthenogens for which Fisher’s exact test has been used to determine p and odds ratio, in comparison to the genomic background (n = 15,972). [file 40851_2022_197_MOESM2_ESM.jpg]
